# The clinical value of ficolin-3 gene polymorphism in rheumatic heart disease. An Egyptian adolescents study

**DOI:** 10.1186/s13104-021-05450-w

**Published:** 2021-01-26

**Authors:** Maher H. Gomaa, Emad Gamil Khidr, Ahmed Elshafei, Hala S. Hamza, Aya M. Fattouh, Ahmed A. El-Husseiny, Ahmed Aglan, Mahmoud Gomaa Eldeib

**Affiliations:** 1 Department of Biochemistry and Molecular Biology, Faculty of Pharmacy, Al-Azhar University, Cairo, Egypt; 2 Department of Pediatrics, Kasr Al-Ainy School of Medicine, Cairo University, Cairo, Egypt

**Keywords:** Rheumatic fever, Rheumatic heart disease, *FCN3* gene polymorphism, Ficolin-3

## Abstract

**Objective:**

Ficolin-3 is one of the innate immunity molecules that was thought to play a pivotal role in *Streptococcus pyogenes* autoimmunity and its complications; rheumatic fever (RF) and rheumatic heart disease (RHD). We aimed to disclose if there is an association between ficolin-3 (*FCN3)* gene polymorphisms (rs4494157 and rs10794501) and RF with or without RHD for the first time in Egyptian adolescents.

**Results:**

Serum ficolin-3 level was significantly elevated in patients suffering from RF with and without RHD in comparison with control. Regarding *FCN3* gene (rs4494157) polymorphism, a significant correlation was found between the A allele and the susceptibility to RF with or without RHD (OR = 2.93, *P* = 0.0002 and OR = 2.23, *P* = 0.008 respectively). Besides, AA homozygous genotype showed a significant association with RHD risk (OR = 3.47, *P* = 0.026). Patients carrying the A allele (CA + AA) had significantly higher serum ficolin-3 than those carrying the CC genotype (*P* ˂ 0.0001). While the frequency of (rs10794501) polymorphism revealed no significant differences between the controls and RF patients with or without RHD (OR = 1.43, *P* = 0.261 and OR = 1.48, *P* = 0.208 respectively).

## Introduction

Rheumatic fever (RF) is a consequence of recurrent group-A *Streptococcus pyogenes* (GAS) pharyngitis as an immune-mediated complication in genetically susceptible individuals [1, 2]. Repeated or severe episodes of RF lead to permanent harm to the heart valves, with subsequent development of rheumatic heart disease (RHD). A high prevalence (31 per 1000 children) of RHD in school-age children in Egypt and other African countries was reported [3].

Rheumatic fever and RHD are multifactorial disorders that involve multiple environmental and genetic factors [4]. The pathogenesis of RF and its sequel, particularly RHD is strongly dependent on autoimmunity. Autoantibodies produced from molecular mimicry between proteins of heart tissue and GAS mediate tissue damage. It was found that GAS molecules as N-acetyl-β-D-glucosamine (GlcNAc) and M protein display a cross-reactivity with valves and myocellular contractile proteins of the host. As one of the pathogen associated molecular patterns and a dominant antigen in GAS cell wall, GlcNAc serves as a target for identification by ficolins [2, 5–7].

Ficolins are group of patterns recognizing proteins that activate the complement system. Currently, they are classified into three types: ficolin-1 (M ficolin), ficolin-2 (L ficolin), and finally ficolin-3 (H ficolin) [7]. Ficolin-3 is mainly synthesized in both liver and lungs, representing the most abundant circulating ficolins [8]. Serum ficolin-3 has been reported to be widely variable among healthy individuals, which may be attributed to the difference in the genetic makeup [9–11]. Functionally, like other ficolins, ficolin-3 has been shown to activate the complement system [12, 13].

Ficolin-3 is encoded by the *FCN3* gene which is located on chromosome 1p36 and contains eight exons. Few studies showed an association between ficolin genes polymorphisms and infectious and autoimmune diseases [14–19]. Additionally, it was reported that there are associations between gene polymorphisms of ficolin-1 [20] and ficolin-2 [21], 22 with RF. However, the impact of ficolin-3/*FCN3* gene polymorphisms on RF and RHD is currently obscure.

Despite being a preventable disease, RHD may proceed silently until patients are presented as debilitating heart failure cases. In this case, surgery is the only possible choice for treatment [23], and deadly outcomes ultimately occur [24]. Indeed, there is a need for a predictive tool or a marker for early detection of RF/RHD and preventing their progression, as well as facilitating early medical follow-up.

This study was designed to investigate for the first time the association of two *FCN3* gene polymorphisms (rs4494157 and rs10794501) as well as serum ficolin-3 levels with the susceptibility of RF and RHD development in Egyptian adolescents.

## Main text

### Methods

This study was performed on 240 Egyptian subjects locating in Cairo that were classified into three groups. The first group consisted of 80 RF patients without RHD. The second group included 80 RF patients with RHD. While in the third group, eighty apparently healthy volunteers matching with the patients for age, sex, ethnic and geographic origin were selected as controls. The demographic data of individuals enrolled in the study were presented in Table 1. RF patients were recruited from the Cardiology Outpatient Clinic, Children Hospital, Cairo University, Egypt. RF diagnosis was carried out based on the modified Jone’s criteria [25]. A free written informed consent form was signed by parents of both controls and patients. Approval of this study by the ethical committee of Children Hospital, School of Medicine, Cairo University, Egypt was obtained. Patients with any infections, acute RF, infective endocarditis, or any other inflammatory disorders were excluded from this study. All enrolled patients had a clinical history of RF. The presence of mitral valve regurge in patients with RHD was confirmed by an echocardiogram.

**Table 1:** Demographic data of all studied groups

Characteristics		Controls (n. = 80)	RF (n. = 160)	
			Without RHD (n. = 80)	With RHD (n. = 80)
Gender	Female n. (%)	48 (60.0)	46 (57.5)	48 (60.0)
	Male n. (%)	32 (40.0)	34 (42.5)	32 (40.0)
Age (years) mean ± SE Age range		15.2 ± 0.29 13–20	14.5 ± 0.43 9–18.5	14.3 ± 0.33 9–18.5

A venous blood sample was withdrawn from all subjects and divided into two aliquots; one for serum separation and determination of serum ficolin-3 levels by enzyme-linked immunosorbent assay (ELISA) using commercial kits (Ray Bio Kit Inc., Georgia, USA) based on manufacturer’s instructions and recommendations, While the 2nd one for *FCN3* gene polymorphisms (rs4494157 and rs10794501) typing (Additional file 1).

Extraction of Genomic DNA (gDNA) from the whole blood sample was performed using Gene JET™ Whole Blood DNA Purification Mini Kit (Thermo Fisher Scientific Inc., USA). Polymorphisms at (rs4494157 and rs10794501) in the *FCN3* gene were typed by real-time polymerase chain reaction (RT-PCR) utilizing TaqMan® allele discrimination assay (Applied Biosystems, CA, USA).

GraphPad Prism 6.2 (GraphPad Software, San Diego, USA) was utilized to perform the statistical analysis of our data. Normality distribution of variables was checked using D’Agostino-Pearson Omnibus test, where normally distributed variables were presented as mean ± SE while we used median (inter-quartile range) to represent the skewed distributed variables. Kruskal–Wallis test was used to compare between all groups followed by Dunn’s test. Genotypes distribution for the polymorphism was checked for deviation from the Hardy–Weinberg equilibrium and any deviations between observed and expected frequencies were examined for detection of significance depending on the χ^2^ test. Besides, odds ratios (ORs) and 95% confidence intervals (CIs) were calculated. A *P*-value < 0.05 was considered statistically significant.

## Results

The genotypic and allelic results of *FCN3* single nucleotide polymorphism (SNP) (rs4494157) analysis for the studied groups were listed in Table 2. This genotype distribution showed no deviation from Hardy–Weinberg equilibrium (*P* > 0.05). The genotypic distribution revealed a higher frequency of the heterozygous CA only and CA/AA genotypes in RF patients with and without RHD when compared to controls. Moreover, the homozygous variant AA genotype showed higher frequency in RF with RHD as compared to controls. Additionally, a higher frequency of the A allele was observed in RF patients with and without RHD when compared to the controls.

**Table 2:** Distribution of *FCN3* genotypes (rs4494157 and rs10794501) in controls and patients with RF

	Control n (%)	RF without RHD n (%)	RF with RHD n (%)	OR (95% CI)	*P*
rs4494157					
Genotype distribution					
CC	64 (80)	49 (61.25)	43 (53.75)		
CA	10 (12.5)	20 (25)	23 (28.75)	2.61 (1.12–6.08)^a^ 3.42 (1.48 -7.91) ^b^	0.026^a*^ 0.005^b*^
AA	6 (7.5)	11 (13.75)	14 (17.5)	3.47 (1.24–9.74) ^b^	0.026^b*^
CA/AA	16 (20)	31 (38.75)	37 (46.25)	2.53 (1.24–5.14) ^a^ 3.44 (1.70–6.95) ^b^	0.016 ^a *^ 0.0007 ^b *^
Allele frequencies					
C	138 (86.25)	118 (73.75)	109 (68.12)		
A	22 (13.75)	42 (26.25)	51 (31.88)	2.23 (1.26–3.95)^a^ 2.93 (1.68–5.14) ^b^	0.008^a*^ 0.0002^b*^
rs10794501					
Genotype distribution					
TT	58 (72.5)	51 (63.75)	54 (67.5)		n.s
TA	17 (21.25)	21 (26.25)	16 (20)		
AA	5 (6.25)	8 (10)	10 (12.5)		
TA/AA	22 (27.5)	29 (36.25)	26 (32.5)		
Allele frequencies					
T	133 (83.1)	123 (76.9)	124 (77.5)		n.s
A	27 (16.9)	37 (23.1)	36 (22.5)		

OR, odds ratio; CI, confidence intervals; RF, rheumatic fever; RHD, rheumatic heart disease; ^a^RF patients without RHD vs. control; ^b^RF patients with RHD vs. control; *Statistically significant different at *P* < 0.05 using Fisher exact test, n.s., non-significance

The genotype distribution for *FCN3* SNP (rs10794501) showed no deviation from Hardy–Weinberg equilibrium. No statistical significance was detected for the frequency of all genotypes and alleles among all groups, as enlisted in Table 2.

**Fig. 1:**
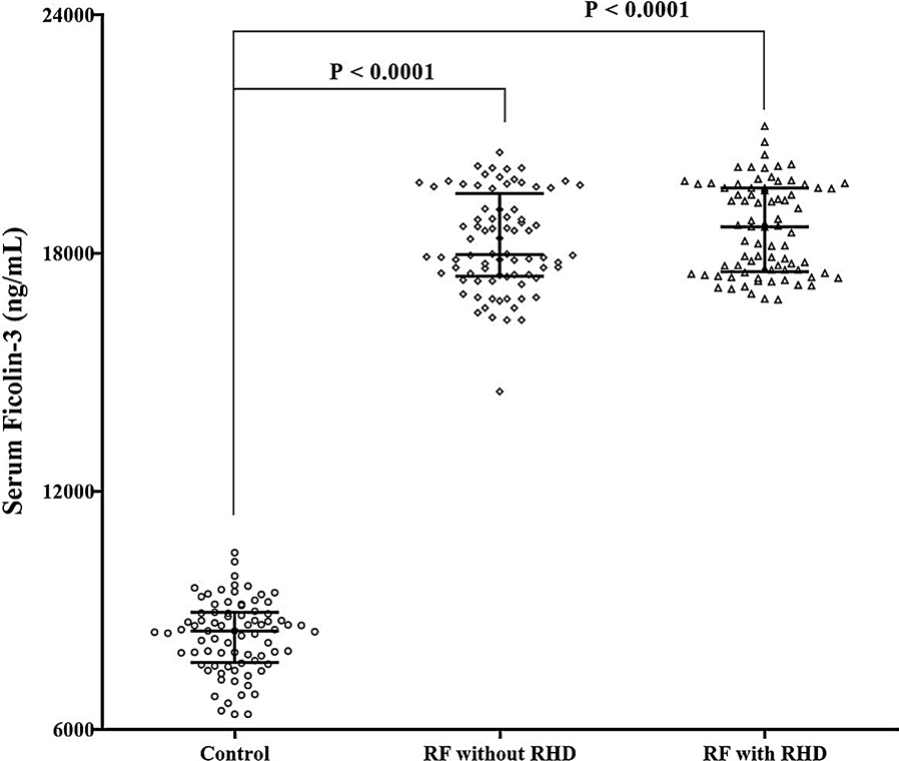
Distribution of Ficolin-3 levels in the investigated groups. Comparisons were made using Kruskal–Wallis test followed by Dunn’s test. Bars indicate median and interquartile values

Serum ficolin-3 levels (ng/mL) were significantly increased in RF patients with and without RHD (18665 (1535–19640)) and (17965 (17420–19503)) respectively Vs controls (8490 (7695–8955)), while there was no significant difference between RF with and without RHD as shown by Fig. 1.

On studying the differences in serum ficolin-3 levels according to the *FCN3* (rs4494157) genotypes in all patients, results revealed that patients carrying the A allele (CA + AA) were associated with significantly higher serum ficolin-3 (Mean ± SE = 18941 ± 128.6) than those carrying the CC genotype (18059 ± 119.2), *P* ˂ 0.0001. However, the classification of the RF group according to the *FCN3* (rs10794501) genotypes showed no significant differences regarding serum ficolin-3 level between TT (18555 ± 115) and TA + AA genotypes (18203 ± 159.7), *P* = 0.111. Thus, no association between ficolin-3 and *FCN3* gene polymorphism (rs10794501) was detected.

## Discussion

Few genetic polymorphisms in the innate proteins that participate in the immune responses have been implicated in RF and RHD vulnerability [26]. Unfortunately, studies on the significance of ficolins in RF and RHD are insufficient, despite their obvious contribution in the activation of the innate immune response through complements and in autoimmunity [27]. Few previous studies on *FCN1* and *FCN2* genes have determined that some polymorphisms of both genes could give a protective role against RF, by empowering bacterial elimination in addition to activation of the expression of these genes leading to an increase in the production of their proteins [20–22].

Up till now, the role of *FCN3* gene polymorphisms in RF and RHD pathogenesis remains unknown. As far as we could possibly know, this is the first study to investigate *FCN3* gene polymorphisms (rs4494157 and rs10794501) together with their related genotypes and levels of serum ficolin-3 in patients suffering from RF and RHD.

In the current study, the significant higher serum level of ficolin-3 in RF patients with and without RHD than the control subjects reflects its role in complements initiation and subsequent pathogenesis of RF and RHD. There have been no reports concerning the relationship between ficolin-3 and RF but several findings are indicating that high levels of ficolin-3 may contribute to the induction of inflammation as in diabetic retinopathy [28], leprosy [29], ovarian cancer [30], acute leukemia [31] and associated with post-operative graft loss in kidney transplantation [32].

Based on the inflammatory progression associated with high levels of ficolin-3, it was assumed that the involvement of ficolin-3 in immune evasion of *Streptococcus pyogenes* in RF and RHD patients is a result of its anti-opsonic response to complements overactivation [33].

Interestingly, lectin pathway activators, including mannose-binding lectin (MBL), both ficolin-1 and ficolin-2 were appeared to bind to *Streptococcus pyogenes* leading to MBL associated serine proteases activation [34]. Although, no direct binding of ficolin-3 on *Streptococcus pyogenes* was found, it is known that the *Streptococcus pyogenes* cell wall contains long polymers of ficolins target; GlcNAc [35] and hence could be a potential ligand for ficolin-3. Therefore, our findings open a new window to study the potential interaction between ficolin-3 and *Streptococcus pyogenes*.

Ficolins are group of proteins with different pattern in tissue expression as well as their immunological roles. The reason beyond the decline in ficolin-1 and ficolin-2 levels in RF patients like what was found in other studies [20–22] may be a result of their consumption on the surface of GAS, in addition to the possibility of transcriptional mutations in their genes that may affect their levels.

As the most abundant ficolins in plasma [8], ficolin-3 level was more elevated even after being utilized in immune reactions against GAS, besides the possible effect of our studied SNPs on its expression. In addition to, the compensatory mechanisms of up-regulation of this protein that resulted from its interaction with GlcNAc as well as complement activation.

Given the (rs4494157), we observed that higher ficolin-3 levels were also associated with certain genotypes of *FCN3* that contain the A allele in intron 7. Interestingly, intron 7 contains CpG islands and enriched for typical modifications of histone that are known to characterize active enhancers [36, 37].

The most important result in this study was related to the *FCN3* A allele (rs4494157). Our finding suggests that this allele may be a risk factor for the progression of RF to its chronic consequences. Thus, *FCN3* A allele carrying patients may be at high risk for recurrent infection, and a higher likelihood to develop RHD. Consequently, early identification, careful monitoring should be given for those patients. Furthermore, clinicians must confirm adherence of those patients to secondary prophylaxis intervention [38]. In fact, secondary prophylaxis adherence of RF patients is typically poor, especially in young people which was perceived as the principal explanation for RF repeats and RHD advancement [38, 39].

What is more, the presence of the C allele in controls in a higher significant pattern than RF patients could show that the presence of the C allele may pose a defensive action against the occurrence of RF and RHD. Moreover, these data propose that the cardiac manifestations development of RF is related to high ficolin-3 levels and its linked genotypes, also, this relationship is a direct result of a certain mechanism related to *FCN3* gene polymorphism but not secondary to the acute phase of GAS infection. This mechanism may involve the recognition of structures on damaged/altered cardiac cells by ficolin-3 that mediates complement activation and increases tissue injury, which may become permanent in the cardiac valves.

## Conclusion

This study suggests that the variant A allele of *FCN3* (rs4494157) is associated with high serum ficolin-3 levels, and the susceptibility to RF and RHD indicating a need for echocardiographic screening, and prophylactic intervention to prevent the disease burden, especially in resources constrained nations.

## Limitations

SNPs selection was dependent on what we have found in the literature. However, there are many SNPs that could be highly prominent in ethnics included in this study.

The sample size was not huge enough to clarify a more extensive picture for *FCN3* genotype distribution among Egyptian adolescents. So, to verify these findings, further larger sample-based studies are recommended in Egyptians and Mediterranean ethnics.

## Supplementary Information

**Additional file 1.** ELISA kit methodology and genetic techniques details.

## Data Availability

The datasets generated and/or analysed during the current study are available in the ClinVar repository, Accession numbers SCV001468298-SCV001468299.
