# Supplementary material for: The clinical value of ficolin-3 gene polymorphism in rheumatic heart disease. An Egyptian adolescents study
Source: BMC Res Notes. 2021 Jan 26;14:36. doi: 10.1186/s13104-021-05450-w (PMC7836457; doi:10.1186/s13104-021-05450-w)
Supplement: Supplementary file 1 — Additional file 1. ELISA kit methodology and genetic techniques details. [file 13104_2021_5450_MOESM1_ESM.docx]

**BMC Research Notes**

RESN-D-20-01451
**Clinical Value of Ficolin-3 Gene Polymorphism in Rheumatic Heart Disease. An Egyptian Adolescents Study**

**ELISA kit methodology:**

Serum ficolin-3 levels in all investigated groups were estimated by enzyme-linked immunosorbent assay using commercial kits (Ray Bio Kit Inc., Georgia, USA).

**Assay Principle:**

This assay employs an antibody specific for human Ficolin-3 coated on a 96-well plate. Standards and samples were pipetted into the wells and Ficolin-3 present in a sample is bound to the wells by the immobilized antibody. The wells were washed, and biotinylated anti-human Ficolin-3 antibody was added. After washing away unbound biotinylated antibody, horseradish peroxidase (HRP)-conjugated streptavidin was pipetted to the wells. The wells were again washed, a tetramethylbenzidine (TMB) substrate solution was added to the wells and color developed in proportion to the amount of Ficolin-3 bound. The Stop Solution changed the color from blue to yellow, and the intensity of the color was measured at 450 nm.

**Assay procedure:**

100 µl standard or sample were added to each well and incubated for 2.5 hours at room temperature then after washing, 100 µl prepared biotin antibody were added to each well and incubated for 1 hour at room temperature. Washing was repeated and 100 µl prepared Streptavidin solution were added and incubated for 45 minutes at room temperature then after washing, 100 µl TMB One-Step Substrate Reagent were added to each well and incubated for 30 minutes at room temperature. Finally, 50 µl Stop Solution were added to each well and color were read at 450 nm immediately.

**Sensitivity of the kit:**

The minimum detectable dose of Human Ficolin-3 was determined to be 0.25 ng/mL.

Intra-Assay CV%: <10%

Inter-Assay CV%: <12%

**Specificity of the kit:**

This ELISA antibody pair detects Human Ficolin-3 only. Other species not determined.

**Unit of measure:** ng/mL.

**Molecular techniques:**

**1- Genomic DNA extraction:**

Genomic DNA (gDNA) was extracted from the whole blood of participants with DNA Blood extraction kits ***(****GeneJET^TM^* Whole Blood Genomic DNA purification Mini Kit, Thermo Scientific, USA***)***.

**Steps of DNA extraction:**

1. Twenty µl of Proteinase K solution was added to 200 µl of whole blood of each sample and mixed by vortexing. Then 400 µl of lysis solution was added and mixed thoroughly by vortexing to obtain a uniform suspension.
2. The mixture was incubated at 56^o^C for 10 minutes in a shaking water bath until the cells were completely lysed.
3. Two hundred µl of 96% ethanol was added and mixed by pipetting.
4. The prepared mixture was transferred to the spin column and centrifuged for 1 minute at 8000 rpm then the collection tube containing the flow-through solution was discarded and the column was placed into a new 2 ml collection tube.
5. Five hundred µl of the prepared wash buffer I was added and centrifuged for 1 minute at 10,000 rpm. The flow-through was then discarded and the column was placed back into the collection tube.
6. Five hundred µl of the prepared wash buffer II was added to the column and centrifuged for 3 minutes at maximum speed 14,000 rpm.
7. The collection tube was emptied and the purification column was placed back into the tube then the column was re-spun for 1 minute at 14,000 rpm.
8. The collection tube containing the flow-through solution was discarded and the column was transferred to a sterile 1.5 ml microcentrifuge tube.
9. Fifty µl of elution buffer was added to the center of the column
   membrane to elute gDNA, incubated for 2 minutes at room temperature (25 ^o^C) and centrifuged for 1 minute at 10,000 rpm.
10. All purified gDNA for each sample was eluted into the tube and the purification column was discarded.

**2-** Assessment of DNA concentration and purity

The gDNA concentration and purity (A260/280) were assessed for each sample before its use in genotyping assays using *NanoDrop*™ 2000 UV-Vis spectrophotometer (Thermo Scientific, Wilmington, *Delaware,* USA).

**3-**Amplification and genotyping

The gDNA was amplified by TaqMan^®^ Universal PCR Master Mix II supplied by *Applied Biosystems,* California, USA.

Predesigned TaqMan^®^ SNP Genotyping Assay kits were used for both studied SNPs (rs4494157, ID[C__11597746_10](https://www.thermofisher.com/order/genome-database/details/genotyping/C__11597746_10?CID=&ICID=&subtype=)) and (rs10794501, ID[C__31148020_10](https://www.thermofisher.com/order/genome-database/details/genotyping/C__31148020_10?CID=&ICID=&subtype=)) and were supplied by *Applied Biosystems*, California, USA.

For rs4494157, the Context Sequence [VIC/FAM]: TTATAGAGGAGGAAACTGAGGCTCA[G/T]TGGAGGGGTAGGACTTGCCCAAGGT

While, for rs10794501, the Context Sequence [VIC/FAM]:

GCACTCAACTTAAGGTCTTTTCAAA[A/T]GGCACCTCCTCTGTGAAGCCCTCAT

Further details are restricted to Applied Biosystems and cannot be provided. Most of the purified DNA samples had concentration of 20 ng/µl which was the recommended DNA concentration per PCR well for TaqMan® SNP assay and the samples with DNA concentration above 20 ng/µl were diluted with DNase-free water while the samples with DNA concentration less than 20 ng/µl, more volume were taken to deliver a final DNA mass for all DNA samples of 20 ng/µl before transferring it to the optical reaction plate for thermal cycling.

One No Template Control sample of DNase-free water was used in each plate to orient the VIC-dye and/or FAM-dye clusters to an origin and to enable the detection of DNA contamination on a given plate.

The reaction mixture for each assay was prepared before transferring it to the optical reaction plate for thermal cycling. This reaction mixture was made from 0.5 µl of (40X TaqMan® SNP (rs4494157 or rs10794501) genotyping assay) and 10 µL of (2X TaqMan® universal PCR master mix) without addition of DNAase free water and DNA sample.

The total volume from the reaction mixture components required for the 48-well plate of the PCR was calculated by multiplying the needed volume of each component for each well (0.5 µL + 10 µL) by 53 to avoid pipetting error, and was prepared and pipetted into a sterile microcentrifuge tube. The tube was closed and thoroughly mixed by swirling it and then centrifuged briefly to spin down the contents and to eliminate any air bubbles from the reaction mix.

The final reaction volume per well was 20 µL which consisted of:

1. TaqMan^®^ Universal Master Mix II, 2X (10 µl).
2. TaqMan^®^ SNP (rs4494157 or rs10794501) Genotyping Assay, 40X (0.5 µl).
3. DNA template + DNase-free water (9.5 µl).

**Procedure**

1. The gDNA samples (~ 20 ng per well) were applied to the optical reaction plate and the reaction mixture was added to the DNA samples in the plate and then the DNAase free water was added in a volume that complete the final reaction volume to 20 µl.
2. The plate was mixed thoroughly to avoid stratification of the reagents and/or air bubbles in the well and all the wells were inspected for uniformity of volume and the plate was covered using MicroAmp^®^ Optical Caps then vortexed.
3. The reaction plate was centrifuged to spin down the contents and eliminate any air bubbles and was loaded into the thermal cycler of real-time PCR (Step One Real-Time PCR, Applied Biosystems, CA, USA)

The thermal cycling conditions were performed as following:

| System | Polymerase activation | PCR | |
| --- | --- | --- | --- |
|  | Hold | Cycle (45 cycles) | |
|  |  | Denature | Anneal/extend |
| Temp. (°C) | 95 | 95 | 60 |
| Time (mm:ss) | 10:00 | 00:15 | 1:00 |

The system software records the results of the genotyping run on a scatter plot of Allele 1 versus Allele 2 for each SNP.
